# Subthreshold Micropulse Laser vs. Conventional Laser for Central Serous Chorioretinopathy: A Randomized Controlled Clinical Trial

**DOI:** 10.3389/fmed.2021.682264

**Published:** 2021-07-16

**Authors:** Lijun Zhou, Kunbei Lai, Ling Jin, Chuangxin Huang, Fabao Xu, Yajun Gong, Longhui Li, Zhe Zhu, Lin Lu, Chenjin Jin

**Affiliations:** ^1^State Key Laboratory of Ophthalmology, Zhongshan Ophthalmic Center, Sun Yat-sen University, Guangzhou, China; ^2^Department of Medicine, Herbert Irving Comprehensive Cancer Center, Columbia University, New York, NY, United States

**Keywords:** subthreshold micropulse laser, subretinal fluid, central foveal thickness, central serous chorioretinopathy, conventional laser photocoagulation

## Abstract

**Purpose:** To investigate the effectiveness and safety of 577-nm subthreshold micropulse laser (SML) on acute central serous chorioretinopathy (CSC).

**Methods:** One hundred and ten patients with acute CSC were randomized to receive SML or 577-nm conventional laser (CL) treatment. Optical coherence tomography and best-corrected visual acuity (BCVA) were performed before and after treatment.

**Results:** At 3 months, the complete resolution of subretinal fluid (SRF) in 577-nm SML group (72.7%) was lower than that in CL group (89.1%) (Unadjusted RR, 0.82; *P* = 0.029), but it was 85.5 vs. 92.7% at 6 months (unadjusted RR, 0.92; *P* = 0.221). The mean LogMAR BCVA significantly improved, and the mean central foveal thickness (CFT) significantly decreased in the SML group and CL group (all *P* < 0.001) at 6 months. But there was no statistical difference between the two groups (all *P* > 0.05). In the SML group, obvious retinal pigment epithelium (RPE) damage was shown only in 3.64% at 1 month but 92.7% in the CL group (*P* < 0.001).

**Conclusions:** Although 577-nm SML has a lower complete absorption of SRF compared with 577-nm CL for acute CSC at 3 months, it is similarly effective as 577-nm CL on improving retinal anatomy and function at 6 months. Importantly, 577-nm SML causes less damage to the retina.

## Introduction

Central serous chorioretinopathy (CSC) is a common macular condition affected mainly in middle-aged patients. It is characterized by a serous neuroepithelium detachment with or without retinal pigment epithelium (RPE) detachment (1). The acute CSC is considered self-limited and usually resolves spontaneously within 3 to 6 months (2, 3). Therefore, observation is often recommended as the current care for acute CSC (1). However, spontaneous resolution does not always occur, and 30–50% of the patients with CSC experienced recurrence. Even 5% of patients progressed to chronic CSC, resulting in permanent damage in visual acuity (4–6). What's more, the outer nuclear layer and photoreceptor could be injured as long as subretinal fluid (SRF) is present (7). Based on the above conditions, some proper treatments for acute CSC are reasonable.

Photodynamic therapy (PDT) and conventional laser (CL) are the mainly proved methods. Although PDT is effective for CSC, it causes ischemia and atrophy of the choroid (8). Besides, it is off-label and expensive for most patients, particularly in developing countries. CL can seal the leakage and accelerate the resolution of SRF, but it is not favorable for the leakage close to the fovea because it usually leads to retinal scars and scotoma (9, 10), which significantly impaired visual function. Therefore, less or non-damage treatment is need.

A 577-nm subthreshold micropulse laser (SML) is a reliable and cost-effective treatment. Furthermore, the 577-nm wavelength is yellow light and is outside the absorption spectrum of retinal xanthophylls, which potentially facilitates treatment close to the fovea (11). Recently, it has been reported that SML treatment is useful for the CSC without apparent retinal damage (12, 13) and better than observation for acute CSC (14). However, the patients in previous studies are almost chronic CSC (12, 15), and there is no prospective report that compared the efficacy of the SML with CL for acute CSC. Therefore, we conducted a clinical trial to compare the effectiveness of 577-nm SML with a 577-nm CL to treat active acute CSC.

## Materials and Methods

### Study Design

This was a single-center, randomized, controlled trial of 577-nm SML vs. 577-nm CL to treat acute CSC, which was registered on ClinicalTrials.gov (identifier: NCT02784665). The study was carried out at Zhongshan Ophthalmic Center (ZOC), Sun Yat-sen University in China, from June 2016 to March 2018. Patients were randomized at a ratio of 1:1 into the 577-nm SML group and 577-nm CL group by block randomization, with a block size of 10. The randomization sequence was generated using a computerized randomization stable. All subjects were masked to the treatment allocation groups and gave informed consent before treatment. The study was adhered to the tenets of the Declaration of Helsinki and approved by the Ethics Committee of ZOC.

### Study Population

Acute CSC was defined as persistent SRF for <6 months. The following inclusion criteria were fulfilled: patients between 18 and 55 years of age, first episode, visual symptoms related to CSC for at least 4 weeks, active leakage away from foveal (more than 300 μm) on fundus fluorescein angiography (FFA), abnormal dilated choroidal vasculature on indocyanine green angiography (ICGA), and SRF involving the fovea on spectral-domain optical coherence tomography (SD-OCT). The exclusion criteria were as follows: patients who underwent previous treatment, including PDT, focal laser photocoagulation, intravitreal injection treatment with anti-vascular endothelial growth factor (VEGF); with other fundus diseases such as polypoidal choroidal vasculopathy (PCV), choroidal neovascularization (CNV), other retinal vascular disorders and maculopathies; high myopia; patients receiving the treatment of exogenous corticosteroid systemically; pregnancy; inability to perform relative fundus examination.

### Study Protocol

All patients received complete eye examinations at baseline and followed up at 1, 3, and 6 months after treatment. Best-corrected visual acuity was measured using the decimal chart and was converted to the logarithm of the minimum angle of resolution (LogMAR) for statistical analysis. Fundus angiography (Spectralis HRA + OCT; Heidelberg Engineering, Germany) was performed to determine the leakage spot and to exclude other maculopathies at baseline. SD-OCT and fundus autofluorescein (FAF, Spectralis HRA + OCT; Heidelberg Engineering, Germany) were performed at baseline and each visit. The central foveal thickness (CFT) was defined as the distance from the neurosensory retina's inner surface to the inner surface of the choroid at the fovea measured by OCT. RPE change was assessed using the FFA at the 1-month visit after laser treatment. RPE was categorized into: no RPE damage (no changes at the treatment area), mild RPE damage (focally rough RPE but no obvious laser spot), and obvious RPE damage (presence of clear laser spots).

### Interventions

CL group was treated with a 577-nm laser (Supra 577Y Laser System; Quantel Medical, Clermont-Ferrand, France) using a continuous-wave model with a 100-μm spot diameter, a 0.1-s duration, and 80–120 mW power. A slight gray spot was the endpoint of CL. The micropulse mode of the 577-nm laser was used for the SML group. The micropulse treatment parameters were standardized for all patients, with 100 μm spot size, 200 ms duration, and a 5% duty cycle. The titration was individualized and operated in the normal retina outside the vascular arcades. The titration power was started at 600 mW with a monospot micropulse model and increased gradually until a just visible minimal graying reaction was seen as the threshold burn. Then the laser power was reduced to 50% as the treatment power of SMPL. Titration power ranged from 800 to 1,200 mW. Hence, the treatment power was between 400 and 600 mW. The micropulse laser in a dense pattern overlaid the leakage points, and the number of micropulse spots was <50 in one session. Treatment was performed using the Mainster contact lens (Ocular Instruments, Bellevue, WA, USA).

If the SRF involved in the macular was still present at the 3-month follow-up, the same intervention was repeated. And the SRF of all patients was assessed again as the second outcome at 6-month follow-up.

### Outcome Measures

The primary outcome was the complete absorption rate of the SRF based on the OCT images at 3 months. The secondary outcomes included changes in the BCVA and CFT at every visit and the complete absorption rate of the SRF at the final endpoint (at 6 months). At the same time, we evaluated the damage of RPE based on FAF imaging at 1 month.

### Statistical Analysis

The sample size was designed to enroll 110 patients based on an estimated rate of complete SRF absorption rate at 3-month follow-up of 75% for the 577-nm SML group and 95% for the 577-nm CL group, with 80% power at a 2-sided α of 0.05 to detect a difference between two groups, estimating 20% loss to follow-up. The sample size was calculated with PASS 11 (NCSS Statistical Software, Kaysville, UT).

According to the intention-to-treat analysis principles that all randomized participants were included, we used the last observation carried forward (LOCF) method to impute the missing values at 3 months and the final endpoint. Results were presented as the mean (SD) or median (IQR) for the continuous variables and frequency for categorical variables. Baseline data comparisons between the two groups were performed by a 2-tailed *t*-test for continuous variables with normal distribution, the Wilcoxon rank-sum test for continuous variables with non-normal distribution, the chi-square test, or Fisher exact test for categorical variables. The complete absorption of SRF at a 3-month follow-up, the primary outcome, with a relative risk (RR) and 95% confidence intervals (CIs), was calculated and compared.

For the potential prognostic factors at baseline, variables with *p* < 0.20 level in a simple regression model were added to the multiple regression model. All statistical analysis was performed using SAS statistical software, version 9.4 (SAS Institute Inc., Cary, NC). A *p*-value with <0.05 was considered statistically significant.

## Results

### Baseline Demographic and Clinical Data

**Figure 1:**
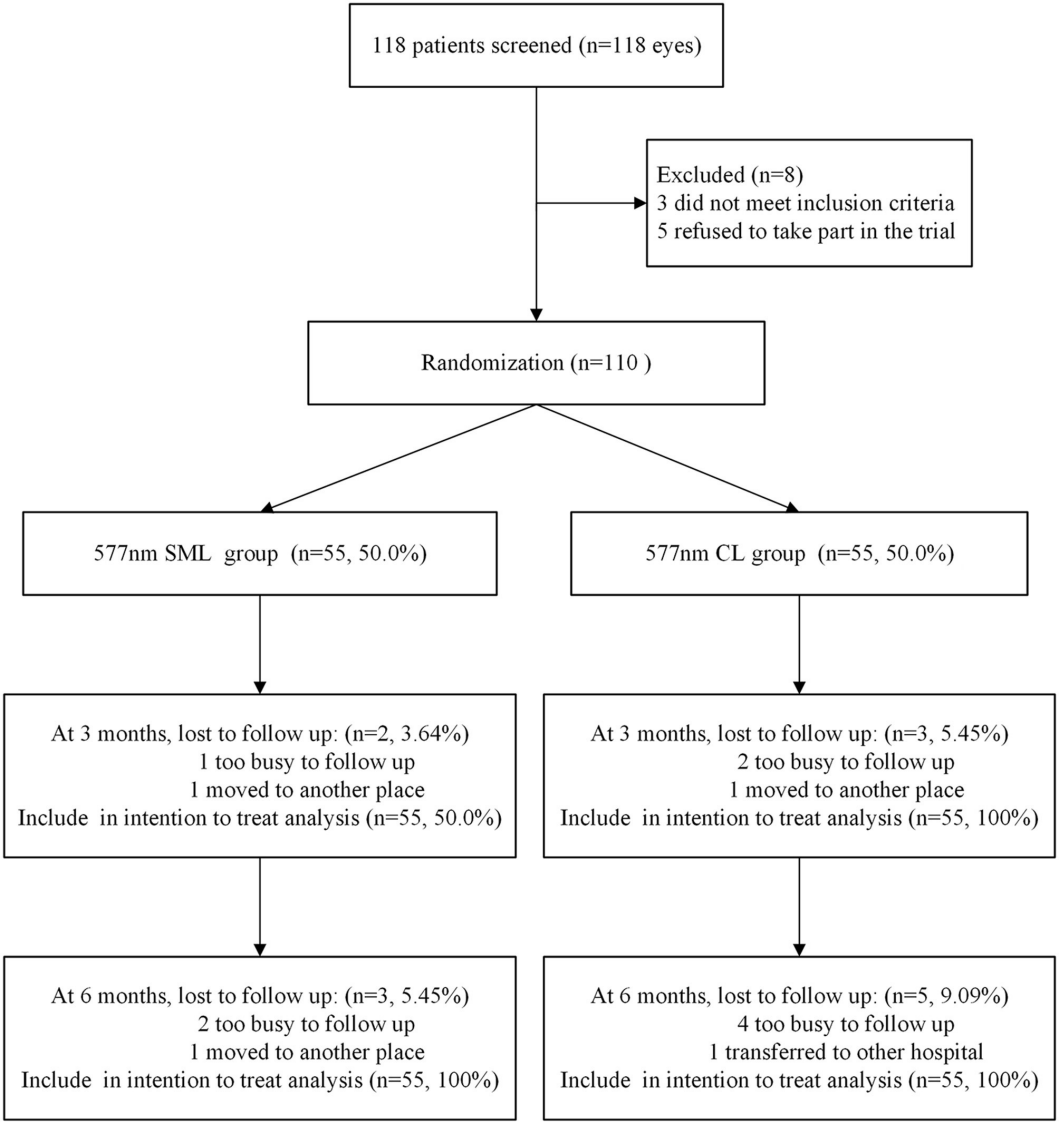
Study design flow chart.

Among 118 eligible patients confirmed as acute CSC with <6-month duration, three (2.54%) did not fit the inclusion criteria, and five (4.24%) declined participation. The remaining 110 patients (93.2%) were enrolled, of which 55 patients (50.0%) were randomly allocated to the 577-nm SML group and 55 patients (50.0%) to the 577-nm CL group. At 3 months, the primary outcome was assessed, and five patients (4.54%) were lost to follow-up: three patients were too busy to follow up, one patient moved to another hospital, and one patient transferred to another place. At 6 months, the secondary outcome was assessed, and eight patients (7.27%) were lost to follow-up: six patients were too busy to follow up, one patient moved to another hospital, and one patient transferred to another place (Figure 1). All missing data were imputed with the LOCF approach for the intention-to-treat analysis.

The baseline demographic and clinical characteristics were balanced between the two groups and are summarized in Table 1. More specifically, there was no significant difference between the SML group and the CL group in the baseline characteristics in terms of the mean BCVA (LogMAR) (0.32 ± 0.21 vs. 0.39 ± 0.22, *P* = 0.113) and mean CFT (474 ± 154 vs. 482 ± 157μm, *P* = 0.780).

**Table 1:** Baseline demographics and clinical characteristics by treatment group (*N* = 110).

**Characteristic**	**577 nm SML group (*n* = 55.50%)**	**577 nm CL group (*n* = 55.50%)**	***P*-value for comparing SML and CL**
Age, mean (SD), years	41.2 (6.00)	41.1 (5.30)	0.894^†^
**Sex, No. (%)**			
Male	47 (85.5%)	46 (83.6%)	0.792^*^
Female	8 (14.5%)	9 (16.4%)	
**Eye, No. (%)**			
Right	29 (52.7%)	33 (60.0%)	0.442^*^
Left	26 (47.3%)	22 (40.0%)	
Duration of symptoms median (IQR), months	2.00 (1.00–4.00)	3.00 (1.00–5.00)	0.519^‡^
BCVA (logMAR), mean(SD)	0.32 (0.21)	0.39 (0.22)	0.113^†^
CFT, mean(SD), μm	474 (154)	482 (157)	0.780^†^

*SD, Standardized deviation; IQR, Interquartile range; BCVA, Best-corrected visual acuity; LogMAR, Logarithm of the minimum angle of resolution; CFT, Central foveal thickness*.

* *Pearson χ2 test*,

† *Independent-sample T-test*,

‡ *Wilcoxon rank-sum test*.

### Resolution of Subretinal Fluid

For the primary outcome at 3-month follow-up, the patients with acute CSC in the SML group had a lower complete resolution rate of SRF [40/55 (72.7%)] compared with patients in the CL group [49/55 (89.1%)], with the significant difference (unadjusted RR, 0.82; 95% CI, 0.68–0.98; *P* = 0.029).

Factors associated with complete resolution of SRF at the 3-month follow-up in multiple regression models included patients in the SML group (RR, 0.78; 95% CI, 0.66–0.93; *P* = 0.005) and baseline BCVA (LogMAR) (RR, 0.44; 95% CI, 0.25–0.16; *P* = 0.004). Other baseline characteristics, including age, sex, duration, and baseline CFT, were not significantly associated with the complete resolution of SRF (Table 2).

**Table 2:** Intention-to-treat analysis of potential determinants of SRF at 3-months follow-up.

**Variable**	**Simple Regression**^†^ **(*****n*** **= 110)**^‡^		**Multiple Regression (*****n*** **= 110)**^‡^	
	**Relative risk (95% CI)**	***P*-Value**	**Relative risk (95% CI)**	***P*-Value**
SML group (CL group as reference)	0.82 (0.68–0.98)	0.033	0.78 (0.66–0.93)	0.005
Age, year	0.99 (0.97–1.01)	0.199	0.99 (0.97–1.01)	0.253
Male sex	0.99 (0.77–1.28)	0.969	–	–
Right eye	1.19(0.98–1.45)	0.077	1.05 (0.86–1.27)	0.657
Duration of symptom, months	0.98 (0.93–1.03)	0.363	–	–
Baseline BCVA (LogMAR)	0.43 (0.25–0.75)	0.003	0.44 (0.25–0.16)	0.004
Baseline CFT, μm	0.99 (0.99–1.01)	0.061	0.99 (0.99–1.04)	0.485

† *Variables with P < 0.20 in the simple regression analysis were included in the multiple regression model. Relative risk and 95% CI were estimated using the generalized linear model with the option of Poisson regression*.

‡ *Included five missing data*.

The patients who were still present SRF involved in the macula at a the 3-month follow-up received the same intervention as the baseline: 14 patients in the SML group and five patients in the CL group. And the SRF resolution of all patients was assessed again as the other outcome at the final endpoint (6-month follow-up). The complete resolution of SRF reached 85.5% (47/55) in the SML group and 92.7% (51/55) in the CL group, but there was no significant difference between the two groups (unadjusted RR, 0.92; 95% CI, 0.81–1.05; *P* = 0.221).

### Changes in Visual Acuity

**Figure 2:**
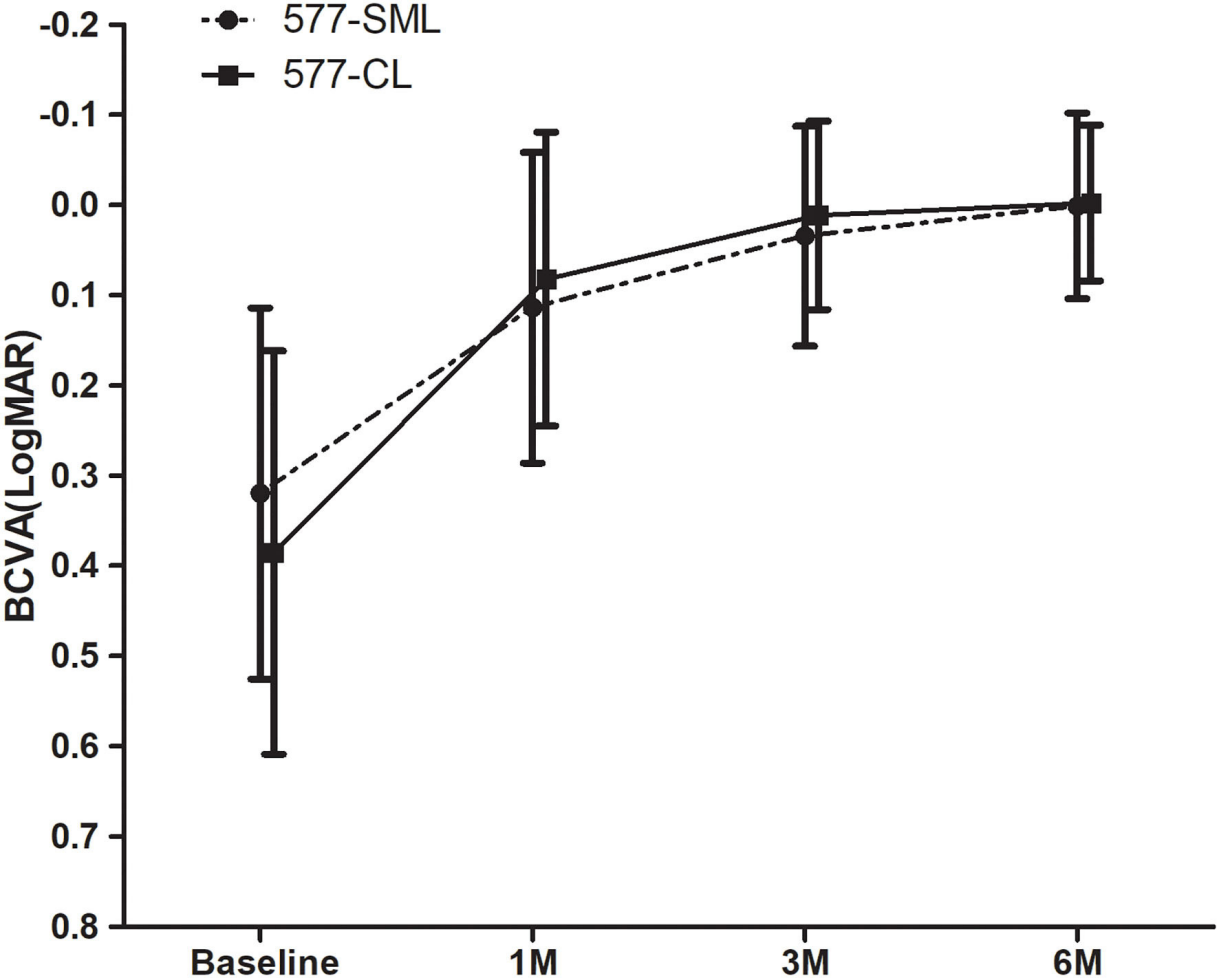
The change of mean LogMAR-BCVA. The BCVA of all patients showed a statistically significant improvement at each visit compared with the baseline in each group (*p* < 0.05). Whereas, there was no significant difference between the 577-nm SML group and the 577-nm CL group (*p* > 0.05). LogMAR, logarithm of the minimum angle of resolution; BCVA, best-corrected visual acuity. SML, subthreshold micropulse laser. CL, conventional laser.

After treatment, the mean visual acuity (LogMAR) had a statistically significant improvement from baseline to the endpoint in the two groups. The mean BCVA in the 577-nm SML group was 0.11 ± 0.17 at 1 month, 0.03 ± 0.12 at 3 months, and 0.00 ± 0.10 at 6 months, respectively, all of which was significantly improved compared with the visual acuity of the baseline (0.32 ± 0.21) (all *p* < 0.001). In the 577-nm CL group, the visual acuity (LogMAR) was markedly improved from 0.39 ± 0.22 at baseline to 0.08 ± 0.16 at 1 month, 0.01 ± 0.11 at 3 months, and 0.00 ± 0.09 at 6 months (all *p* < 0.001). The change of BCVA (LogMAR) in the SML group was lower than that in the CL group with an unadjusted difference (mean, 0.09; 95% CI, 0.02–0.17; *p* = 0.017). However, there was no statistical difference concerning the change of BCVA (LogMAR) at the final endpoint between the two treatment groups (unadjusted mean difference, 0.07; 95% CI, −0.01 to 0.15; *p* = 0.093) (Figure 2).

### Changes of Central Foveal Thickness

**Figure 3:**
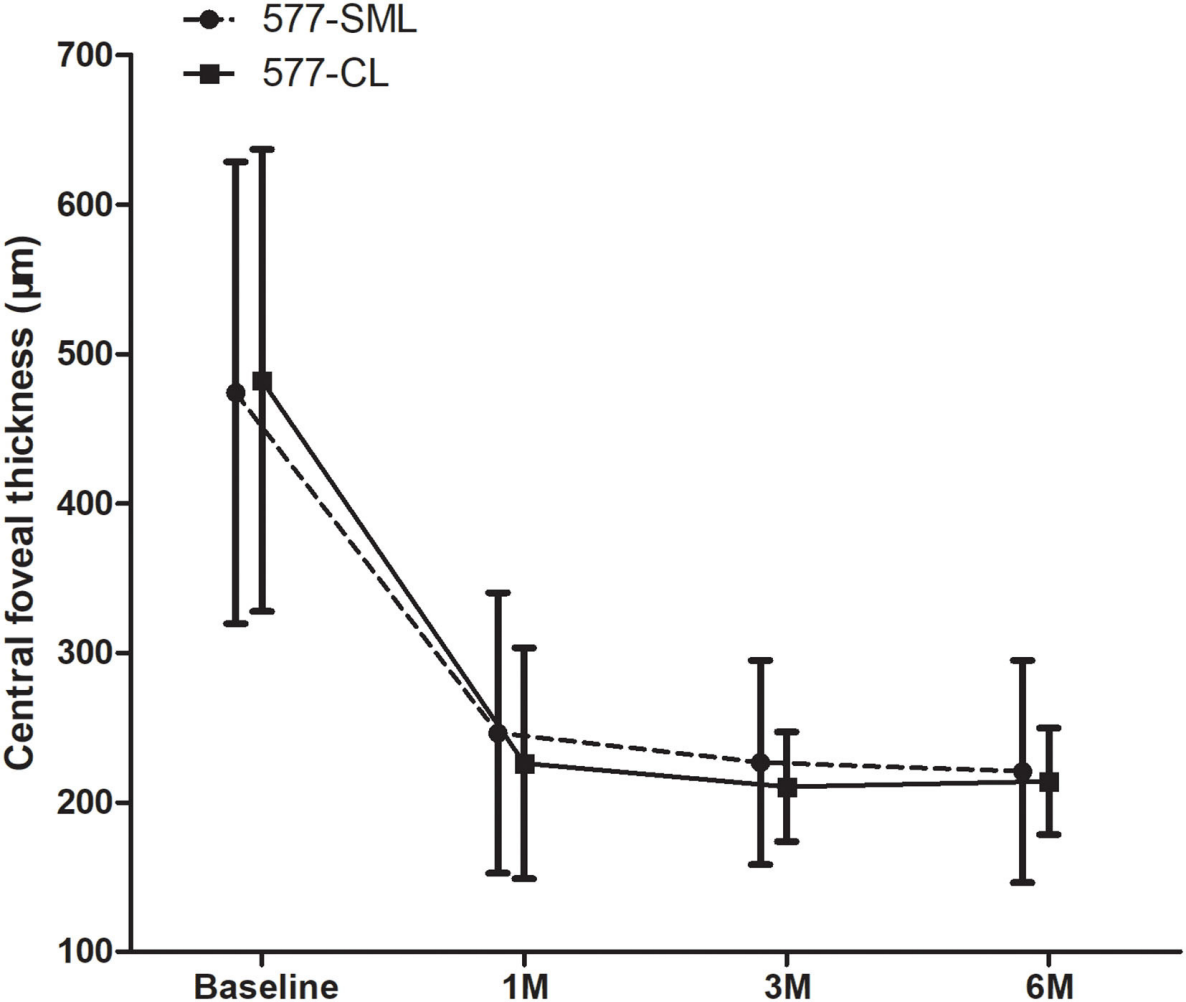
The change of mean CFT during baseline and visits. CFT showed a statistically significant reduction at each visit compared with the baseline in each group (*p* < 0.05). Whereas, there was no significant difference between 577-nm SML group and 577-nm CL group (*p* > 0.05). CFT, central foveal thickness. SML, subthreshold micropulse laser. CL, conventional laser.

In the 577-nm SML group, the mean CFT decreased significantly from 474 ± 154 μm at baseline to 246 ± 93.8 μm at 1 month, 227 ± 68.4 μm at 3 months, and 221 ± 74.4 μm at 6 months (all *p* < 0.001). The mean CFT was 482 ± 155 μm at baseline in the 577-nm CL group and decreased remarkably to 226 ± 77.1 μm at 1 month, 210 ± 36.6 μm at 3 months, and 214 ± 35.6 μm at 6 months (all *p* < 0.001). However, there was no statistical difference for the change of CFT at the 3-month visit (unadjusted mean difference, −24.4; 95% CI, −83.8 to 35.1; *p* = 0.418) and at the 6-month visit (unadjusted mean difference, −14.7; 95% CI, −74.4 to 44.8; *p* = 0.625) between the two treatment groups (Figure 3).

### Safety

**Figure 4:**
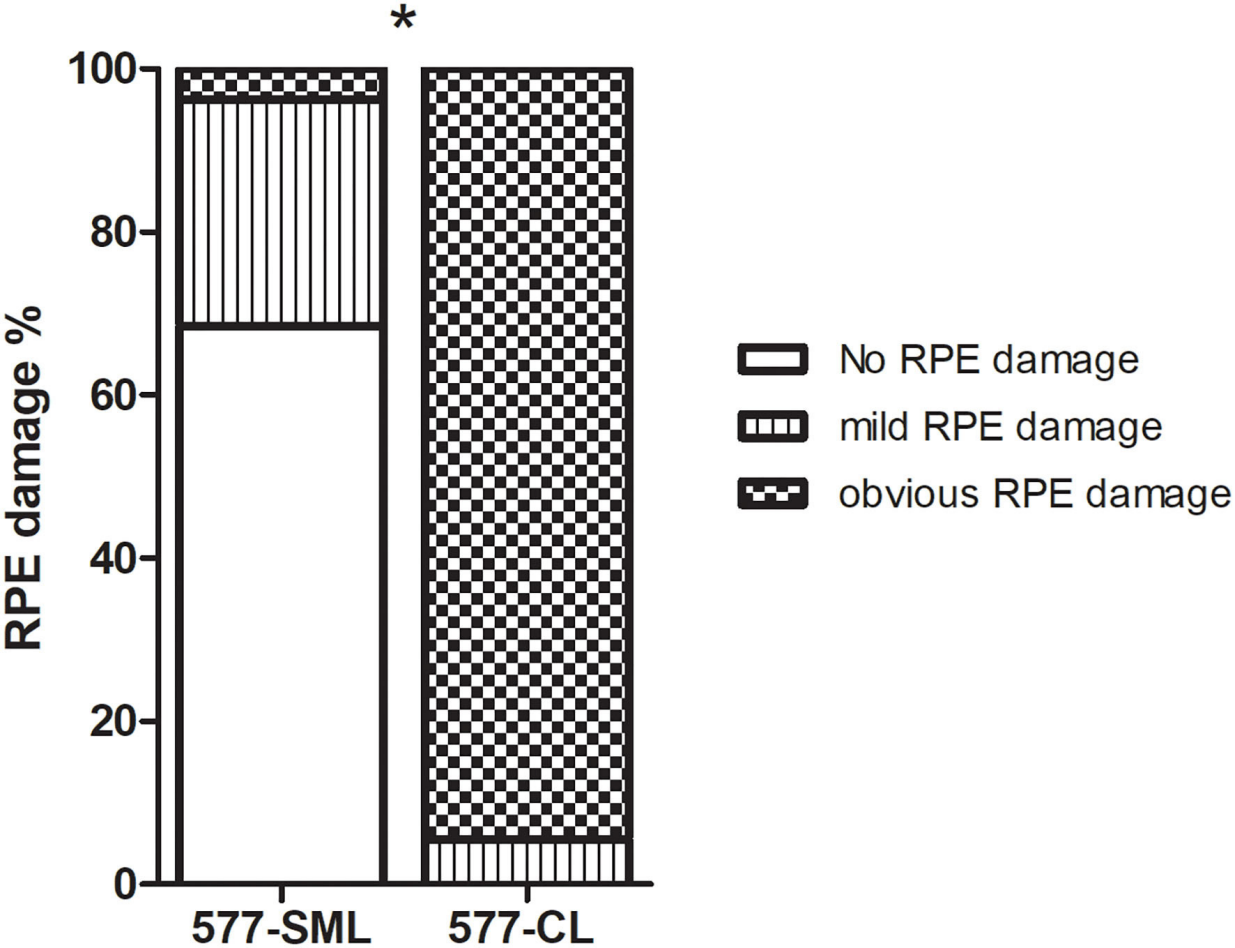
The comparison of eyes with RPE change on FAF imaging at 1 month after treatments in the 577-nm SML group and 577-nm CL group. Error bars represent standard errors of the mean. SML, subthreshold micropulse laser. CL, conventional laser. RPE, retinal pigment epithelium. FAF, fundus autofluorescence. *P < 0.05.

Based on the FAF images, RPE damage was evaluated at a the 1-month follow-up. In the 577-nm SML group, 69.1% (38/55) patients showed no RPE damage, 27.3% (15/55) had mild RPE damage, and only 3.64% (2/55) showed obvious RPE damage. The corresponding data in the 577-nm CL group were 0.00% (0/55), 7.27% (4/55), and 92.7% (51/55), respectively (Figure 4). The patients who received conventional wave laser treatment had more significant RPE damage than the patients who received subthreshold micropulse laser treatment (*p* < 0.001). Besides, during the 6-month follow-up, choroidal neovascularization was not seen on OCT imaging in all patients.

## Discussion

To the best of our knowledge, this study is the first prospective randomized controlled trial on the comparison of 577-nm SML with 577-nm CL for acute CSC. Our study showed that the SRF of patients with acute CSC in the 577-nm SML group had a lower complete absorption rate in the short-term (at 3 months), compared with that in the CL group. But after retreatment, 577-nm SML can reach a similar effect on improving the functional and anatomical outcomes of eyes with acute CSC. Importantly, 577-nm SML scarcely damaged RPE compared with CL.

The conventional laser has been applied to retinal disease for many years, and it works by thermal energy to the RPE (16). Nevertheless, except for the proven effect, it has inherent adverse effects that often destroy the adjacent tissue, such as the inner retina and photoreceptors, due to thermal energy conduction. Therefore, it is not suitable for subfoveal and juxtafoveal leaks (1, 17). In the present study, we showed similar findings of the CL treatment, as reported previously. In the 577-nm CL group, SRF was absorbed entirely in 92.7% (51/55) of patients, and CFT decreased prominently from 482 ± 15 μm at baseline to 214 ± 35.6 μm 6 months (*p* < 0.001). Accordingly, visual acuity improved significantly from 0.39 ± 0.22 LogMAR at baseline to 0.00 ± 0.09 LogMAR (*p* < 0.001) after treatment. But evident laser scar (RPE damage) was seen in 51 (92.7%) patients, significantly higher than that of 577-nm SML.

Minimizing the adverse reactions of CL treatment, new modalities have emerged over the years (18). SML, described in detail by Dorin in 2003, is a newer method for macular disease, especially in diabetic macular edema and chronic CSC in recent years (19–22). SML is composed of a train of repetitive ultrashort laser pulses that has “on-time” and “off-time” and produces a sublethal cellular thermal effect. The SML mechanism in treating CSC may be due to the activation of RPE biological response rather than merely thermal coagulation of RPE (23). As shown in our study, only 3.64% of eyes had obvious RPE damage after SML treatment, significantly less than CL treatment.

Recent retrospective studies have demonstrated that 577-nm SMPL could achieve the equivalent effect as conventional laser but without RPE damage (13). It has been reported that in chronic CSC, the complete resolution rate of the SRF varied from 33 to 75% in previous studies using SML (15, 21, 24). In our research, the result was better with a complete resolution rate of 72.7% at 3 months after the first treatment and 85.5% at 6 months after retreatment. There might be two reasons for the faster absorption ratio. First, patients in previous studies were chronic CSC and might have diffuse damaged RPE that would slow down the absorption of subretinal fluid. While the patients in our study were acute CSC, the RPE reaction to SML would be better. Second, their follow-up was relatively shorter from 2 to 6 weeks but 12–24 weeks in our study. Additionally, our results showed that repeated treatment of SML was available for patients with persistent SRF because of less RPE damage. The findings have shown that SML treatment has fewer side effects, even repeated use.

There is a challenge for SML treatment, especially in power selection. Power titration is an essential step in SML treatment, and it is usually outside the vascular arcades. The method of titration was reported in several clinical studies (12, 15, 25). It was performed in a single spot pattern, and the power was increased gradually until a just visible spot was seen. This power was the threshold burn. Then the laser power was reduced to 50% for the actual treatment power in our study. However, the real treatment power can't fit any retina because retinal epithelial cells and choroidal melanocytes vary from location. Thus, it was explained that RPE damage was seen in two patients of our study. So, it should be used more judiciously when the leakage was just at the macular fovea. If you do, it is better to use <50% titration power (26).

There were several limitations to our study. First, we cannot assess and compare the long-term effect of 577-nm SML with 577-nm CL due to a half-year follow-up. Additionally, an observation group was absent not to eliminate the spontaneous resolution of SRF after the SML. It was reported that acute CSC was resolved spontaneously for 3–6 months and with 57% complete absorption of SRF in previous studies (2, 3, 27). Our study revealed that the complete resolution rate of SRF had reached 72.7% in the 577-nm SML group and 89.1% in the 577-nm CL group at 3 months. Furthermore, a recent randomized controlled trial has shown that subthreshold micropulse laser is significantly superior to observation for acute CSC (14). Therefore, we believed that the change in our study is likely attributable to laser intervention.

In conclusion, our results revealed that 577-nm SML had a lower complete absorption of SRF for acute CSC than 577-nm CL at the 3-month follow-up. But it reached a similar effect on the improvement of anatomy and retinal function at 6 months between the two groups. The findings suggest that SML works gently compared to the CL. Furthermore, 577-nm SML treatment was safer because it had no apparent damage to RPE and could be used repeatedly, even be applied to the macular disease close to the fovea. Therefore, SML is a potential treatment option for patients with acute CSC by accelerating the absorption of SRF and improving the visual function without evident visible retinal damage.

## Data Availability Statement

The raw data supporting the conclusions of this article will be made available by the authors, without undue reservation.

## Ethics Statement

The studies involving human participants were reviewed and approved by the Ethics Committee of Zhongshan Ophthalmic Center. The patients/participants provided their written informed consent to participate in this study. Written informed consent was obtained from the individual(s) for the publication of any potentially identifiable images or data included in this article.

## Author Contributions

CJ: conceptualization. LZ and KL: methodology and writing—original draft preparation. CH: validation. FX: formal analysis. YG: investigation. LZ and LJ: data curation. ZZ and CJ: writing—review and editing. LLi: supervision. LLu: project administration. All authors contributed to the article and approved the submitted version.

## Conflict of Interest

The authors declare that the research was conducted in the absence of any commercial or financial relationships that could be construed as a potential conflict of interest.
